# Diabetes mellitus and other cardiovascular risk factors in lower-extremity peripheral artery disease versus coronary artery disease: an analysis of 1,121,359 cases from the nationwide databases

**DOI:** 10.1186/s12933-019-0955-5

**Published:** 2019-11-15

**Authors:** Mitsuyoshi Takahara, Osamu Iida, Shun Kohsaka, Yoshimitsu Soga, Masahiko Fujihara, Toshiro Shinke, Tetsuya Amano, Yuji Ikari, the J-EVT and J-PCI investigators

**Affiliations:** 1 The Japanese Association of Cardiovascular Intervention and Therapeutics, 2-20-8, Shinkawa, Chuo-ku, Tokyo, 104-0033 Japan; 2 Department of Diabetes Care Medicine, Osaka University Graduate School of Medicine, 2-2 Yamadaoka, Suita, Osaka 565-0871 Japan; 3 Department of Health Quality Assessment, The University of Tokyo, 7-3-1 Hongo, Bunkyo-ku, Tokyo, 113-8655 Japan; 4 Cardiovascular Center, Kansai Rosai Hospital, 3-1-69 Inabaso, Amagasaki, Hyogo 660-8511 Japan; 5 Department of Cardiology, Keio University School of Medicine, 35 Shinanomachi, Shinjuku-ku, Tokyo, 160-8582 Japan; 6 Department of Cardiology, Kokura Memorial Hospital, 3-2-1 Asano, Kokurakita-ku, Kitakyushu, 802-0001 Japan; 7 Department of Cardiology, Kishiwada Tokushukai Hospital, 4-27-1, Kamoricho, Kishiwada, Osaka 596-8522 Japan; 8 Division of Cardiovascular Medicine, Department of Internal Medicine, Showa University School of Medicine, 1-5-8, Hatanodai, Shinagawa-ku, Tokyo, Japan; 9 Department of Cardiology, Aichi Medical University, 1-1 Yazakokarimata, Nagakute, Aichi 480-1195 Japan; 10 Department of Cardiology, Tokai University, 143 Shimokasuya, Isehara, Kanagawa 259-1193 Japan

**Keywords:** Cardiovascular risk factors, Peripheral artery disease, Coronary artery disease

## Abstract

**Background:**

Lower-extremity peripheral artery disease (LE-PAD) and coronary artery disease (CAD) are both pathologically rooted in atherosclerosis, and their shared clinical features regarding the exposure to cardiovascular risk factors have been emphasized. However, comparative data of the two cardiovascular diseases (CVDs) were so far lacking. The purpose of this study was to directly compare the clinical profile between cases undergoing endovascular therapy (EVT) for LE-PAD and those undergoing percutaneous coronary intervention (PCI).

**Methods:**

Data were extracted from the nationwide procedural databases of EVT and PCI in Japan (J-EVT and J-PCI) between 2012 and 2017. A total of 1,121,359 cases (103,887 EVT cases for critical limb ischemia [CLI] or intermittent claudication and 1,017,472 PCI cases for acute coronary syndrome [ACS] or stable angina) were analyzed. Heterogeneity in clinical profile between CVDs was evaluated using the *C* statistic of the logistic regression model for which dependent variable was one CVD versus another, and explanatory variables were clinical profile. When two CVDs were completely discriminated from each other by the developed model, the *C* statistic (discrimination ability) of the model would be equal to 1, indicating that the two CVDs were completely different in clinical profile. On the other hand, when two CVDs were identical in clinical profile, the developed model would not discriminate them at all, with the *C* statistic equal to 0.5.

**Results:**

Mean age was 73.5 ± 9.3 years in LE-PAD patients versus 70.0 ± 11.2 years in CAD patients (P < 0.001). The prevalence of diabetes mellitus and end-stage renal disease was 1.96- and 6.39-times higher in LE-PAD patients than in CAD patients (both *P* < 0.001). The higher prevalence was observed irrespective of age group. The exposure to other cardiovascular risk factors and the likelihood of cardiovascular risk clustering also varied between the diseases. The between-disease heterogeneity in patient profile was particularly evident between CLI and ACS, with the *C* statistic equal to 0.833 (95% CI 0.831–0.836).

**Conclusions:**

The current study, an analysis based on nationwide procedural databases, confirmed that patient profiles were not identical but rather considerably different between clinically significant LE-PAD and CAD warranting revascularization.

## Background

Lower-extremity peripheral artery disease (LE-PAD) has been increasing in prevalence and incidence [5], and has become a global health care problem. LE-PAD is pathologically rooted in atherosclerosis, as is coronary artery disease (CAD). Major risk factors for LE-PAD include age, sex, smoking, diabetes mellitus, hypertension, dyslipidemia, and renal failure, all of which are parallel to those for CAD [1, 2]. Moreover, the clustering of these cardiovascular risk factors will further increase the risk [3]. Consequently, diabetes mellitus and other cardiovascular risk factors are commonly accumulated not only in CAD patients but also in LE-PAD patients. In addition, LE-PAD patients are at high risk of future coronary events [4], as are CAD patients. Such common features of the two cardiovascular diseases (CVDs) have been repeatedly emphasized when discussing the importance of cardiovascular risk management in an LE-PAD population [1, 2]. On the other hand, in clinical practice, clinical features intuitively appear somewhat different between clinically significant LE-PAD and CAD undergoing revascularization.

Clinical guidelines have recommended cardiovascular risk management in LE-PAD patients, sometimes based on evidence shown in CAD patients, to cover scanty evidence in the field of LE-PAD [1, 2, 6, 7]. However, beneficial effects of an intervention to a cardiovascular risk factor vary with background characteristics [8–12]. It would be essential to uncover how similar or different clinical profiles are between LE-PAD and CAD.

The aim of the current study was to describe the clinical profile of symptomatic LE-PAD patients undergoing endovascular therapy (EVT) in comparison to symptomatic CAD patients undergoing percutaneous coronary intervention (PCI), based on data from nationwide databases obtained during the same time interval.

## Methods

The current study used data obtained from national registry databases in Japan (J-EVT and J-PCI) between January 2012 and December 2017. The J-EVT and J-PCI are the nationwide multicenter registries of EVT and PCI in Japan, respectively, organized by the Japanese Association of Cardiovascular Intervention and Therapeutics. The association obliges interventionalists and their cardiovascular centers to register all EVT and PCI cases in the J-EVT and J-PCI, for application for board certification and renewal. The registered data include clinical diagnosis, treated vessel territories, and baseline patient characteristics (sex, age, smoking, hypertension, dyslipidemia, diabetes mellitus, and end-stage renal disease on dialysis). The data analysis was performed in accordance with the Declaration of Helsinki and was approved by the ethics committee of the Clinical Research Promotion Network Japan.

### Study population

Between 2012 and 2017, 1,450,813 cases (117,697 lower-extremity EVT cases and 1,333,116 PCI cases) were registered in the J-EVT and J-PCI. Of these, 104,471 cases underwent aortoiliac, femoropopliteal, and/or below-the-knee EVT for critical limb ischemia (CLI) or aortoiliac and/or femoropopliteal EVT for intermittent claudication; and 1,022,997 cases underwent PCI for acute coronary syndrome (ACS) or stable angina. Cases for which information on age was missing or was out of the range of 20 to 100 years (n = 5709: 0.3%) were excluded from the present analysis.

### Variable definitions

The definition of patient clinical profile (i.e., cardiovascular risk factors) was as follows: smoking was defined as any history of smoking within the past 1 year. Diabetes mellitus was determined when fasting plasma glucose levels were ≥ 126 mg/dl, casual plasma glucose levels were ≥ 200 mg/dl, hemoglobin A1c levels were ≥ 6.5%, plasma glucose levels 2 h after a 75-g oral glucose tolerance test were ≥ 200 mg/dl, or patients were treated with anti-diabetic medications [13]. Hypertension was determined when systolic blood pressure was ≥ 140 mmHg, diastolic blood pressure was ≥ 90 mmHg, or patients were treated with anti-hypertensive medications [14]. Dyslipidemia was determined when fasting triglyceride levels were ≥ 150 mg/dl, fasting high-density lipoprotein cholesterol levels were < 40 mg/dl, fasting low-density lipoprotein cholesterol levels calculated from the Friedewald equation were ≥ 140 mg/dl, non-high-density lipoprotein cholesterol levels were ≥ 170 mg/dl, or patients were treated with anti-hyperlipidemic medication [15]. End-stage renal disease on dialysis included both hemodialysis and peritoneal dialysis. A total of 1.1% of the study population had missing values on one or more of these cardiovascular risk factors, and in the analysis on patient profile, cases with missing data were excluded in a list-wise manner.

CLI was determined when patients presented lower extremity rest pain or unhealed ulcers/gangrenes due to chronic severe ischemia. For PCI patients, acute myocardial infarction (AMI) and unstable angina were referred to as ACS. AMI was further sub-classified into ST-elevation myocardial infarction (STEMI) and non-STEMI. Since 4.6% of AMI cases had missing information regarding its sub-classification, these cases were excluded in the analysis with ACS cases further divided into STEMI, non-STEMI, and unstable angina, whereas otherwise they were included.

### Statistical analyses

Data are presented as the mean ± standard deviation (SD) for continuous variables and as percentages for categorical variables unless otherwise indicated. A *P* value < 0.05 was considered statistically significant, and 95% confidence intervals (CIs) are reported where appropriate. The inter-group difference in mean age was examined using Welch’s *t*-test, whereas that of the prevalence of cardiovascular risk factors was evaluated with the odds ratio. The prevalence of cardiovascular risk factors by age was estimated using a logistic regression model with a cubic spline function.

The likelihood that two arbitrary cardiovascular risk factors were clustered in a patient with a CVD was evaluated in terms of the odds ratio of the two variables, and comparison between CVDs was expressed as the fold difference. Since the inter-CVD difference was expected to be confounded by age, the analysis was performed after the age distributions of respective CVDs were adjusted to a normal distribution of 70 ± 10 years, with outliers of ± 3SD (i.e., 40–100 years) excluded. The 95% CIs of odds ratios and their inter-CVD fold differences were estimated by 2000-time bootstrap resampling.

Heterogeneity in patient profile between CVDs was evaluated with the *C* statistic of the regression model for the CVDs by patient profile. The regression models were developed using a logistic regression model for which dependent variable was one CVD versus another, and explanatory variables were age, cardiovascular risk factors, and their second-order interaction terms. When two CVDs were completely discriminated from each other by the developed model, the *C* statistic (discrimination ability, also known as the area under the receiver operating characteristic curve) of the model would be equal to 1, indicating that the two CVDs were completely different in clinical profile. On the other hand, when two CVDs were identical in clinical profile, the developed model would not discriminate them at all, with the *C* statistic equal to 0.5. The *C* statistic was thus used as an indicator of the degree of heterogeneity in clinical profile between CVDs.

Since practice might change among cardiovascular centers, we additionally performed a series of the investigations with adjustment for institution, using (generalized) linear mixed models in which the inter-institution variability was entered as the random effects. The adjusted inter-CVD difference in mean age was examined using the linear mixed model, whereas the generalized linear mixed model with a logit-link function was adopted to explore the adjusted inter-CVD difference in the prevalence of cardiovascular risk factors, as well as their association with a cubic spline of the age variable. Odds ratios for cardiovascular risk clustering and their inter-CVD fold differences were also estimated using the generalized linear mixed model with a logit-link function. In the model, age was further entered as the fixed effects, for its adjustment. It was expected that a regression coefficient in a function of a variable for another, from which the corresponding odds ratio and fold difference were derived, would not be equivalent to that in a function of the latter for the former. We therefore calculated odds ratios and inter-CVD fold differences from these two regression models. The *C* statistic as an index of inter-CVD heterogeneity was calculated for the estimates derived from the generalized linear mixed model with a logit-link function. During the calculation, the estimates from the developed regression model were obtained with the random effects excluded, to avoid overfitting by the inter-institution variability.

All statistical analyses were performed using R version 3.6.0 (R Development Core Team, Vienna, Austria).

## Results

A total of 1,121,359 patients undergoing EVT (*n* = 103,887; 9.3%) or PCI (*n* = 1,017,472; 90.7%) were analyzed in the current study. As summarized in Table 1, the clinical profile differed substantially between CAD and LE-PAD patients. LE-PAD patients were older than CAD patients (73.5 ± 9.3 years versus 70.0 ± 11.2 years). Diabetes mellitus and end-stage renal disease on dialysis were more prevalent in LE-PAD versus CAD patients, by 1.96 and 6.39 times, respectively. This contrast was obvious when CLI and ACS patients were compared (prevalence higher in CLI patients by 3.12 and 18.7 times, respectively). Furthermore, their high prevalence in LE-PAD, especially in CLI, was more marked when compared to STEMI instead of ACS, and when focused on distal lesions (Additional file 1: Tables S1 and S2).

**Table 1:** Clinical profile in patients with CAD and LE-PAD

	Age (years)	Age ≥ 75 years	Male sex	Smoking
CAD	70.0 ± 11.2 (Ref)	37.9% (Ref)	75.1% (Ref)	30.7% (Ref)
LE-PAD	73.5 ± 9.3 (+ 3.5 [3.4–3.6])	48.0% (1.51 [1.49–1.53])	71.6% (0.83 [0.82–0.84])	33.3% (1.13 [1.11–1.14])
ACS	69.4 ± 12.2 (Ref)	37.2% (Ref)	75.2% (Ref)	34.7% (Ref)
SA	70.7 ± 10.1 (+ 1.3 [1.3–1.3])	38.7% (1.06 [1.06–1.07])	75.1% (1.00 [0.99–1.01])	26.6% (0.68 [0.67–0.69])
CLI	74.4 ± 10.1 (+ 5.0 [4.9–5.1])	52.3% (1.85 [1.82–1.89])	65.6% (0.63 [0.62–0.64])	26.5% (0.68 [0.66–0.69])
IC	73.0 ± 8.7 (+ 3.5 [3.5–3.6])	45.2% (1.39 [1.37–1.41])	75.6% (1.02 [1.00–1.04])	37.9% (1.15 [1.13–1.17])

	Hypertension	Dyslipidemia	Diabetes mellitus	End-stage renal disease on dialysis
CAD	73.1% (Ref)	61.1% (Ref)	41.2% (Ref)	5.5% (Ref)
LE-PAD	77.6% (1.28 [1.26–1.30])	48.6% (0.60 [0.59–0.61])	57.9% (1.96 [1.94–1.99])	27.3% (6.39 [6.29–6.49])
ACS	69.8% (Ref)	58.2% (Ref)	37.9% (Ref)	4.3% (Ref)
SA	76.5% (1.40 [1.39–1.42])	64.1% (1.28 [1.27–1.29])	44.6% (1.32 [1.31–1.33])	6.8% (1.64 [1.61–1.66])
CLI	73.0% (1.17 [1.14–1.20])	38.4% (0.45 [0.44–0.46])	65.5% (3.12 [3.05–3.19])	45.5% (18.7 [18.2–19.1])
IC	80.7% (1.81 [1.77–1.85])	55.5% (0.89 [0.88–0.91])	52.7% (1.83 [1.80–1.86])	14.9% (3.91 [3.81–4.01])

Data are mean ± SD (difference [95% CI]) for continuous variables and percentage (odds ratio [95% CI]) for dichotomous variables. LE-PAD was compared to CAD, whereas SA, CLI, and IC were compared to ACS

*CAD* coronary artery disease, *LE-PAD* lower-extremity peripheral artery disease, *ACS* acute coronary syndrome, *SA* stable angina, *CLI* critical limb ischemia, *IC* intermittent claudication

**Fig. 1:**
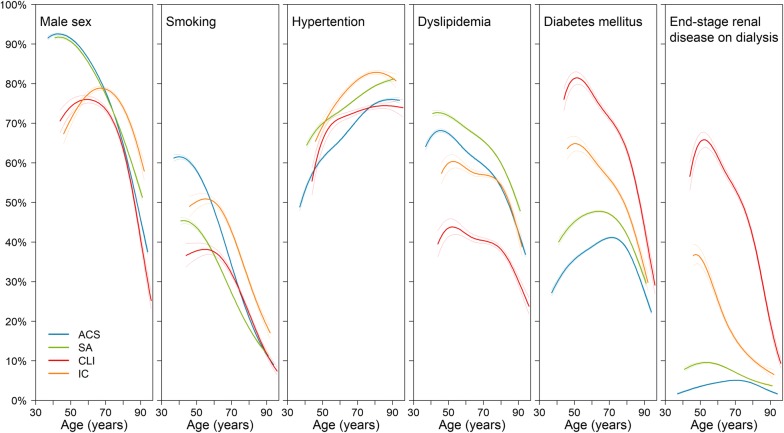
Prevalence of cardiovascular risk factors by age. Solid lines and dotted lines represent estimates and their 95% CIs, corresponding to each age (mean − 3SD to mean + 3SD of age). *ACS* acute coronary syndrome, *SA* stable angina, *CLI* critical limb ischemia, *IC* intermittent claudication

The prevalence of cardiovascular risk factors by age is illustrated in Fig. 1. Irrespective of age, diabetes mellitus and end-stage renal disease on dialysis were most prevalent in CLI, followed by intermittent claudication, stable angina, and ACS cases. In general, the younger generation was more frequently exposed to cardiovascular risk factors, with some exceptions such as hypertension in all CVDs, presenting an almost positive correlation with age, and diabetes mellitus and end-stage renal disease on dialysis in ACS, describing an inverse U-shaped curve. The generally higher prevalence of cardiovascular risk factors in the younger generation indicates in turn that patients exposed to such cardiovascular risk factors were generally younger (Additional file 1: Table S3).

**Fig. 2:**
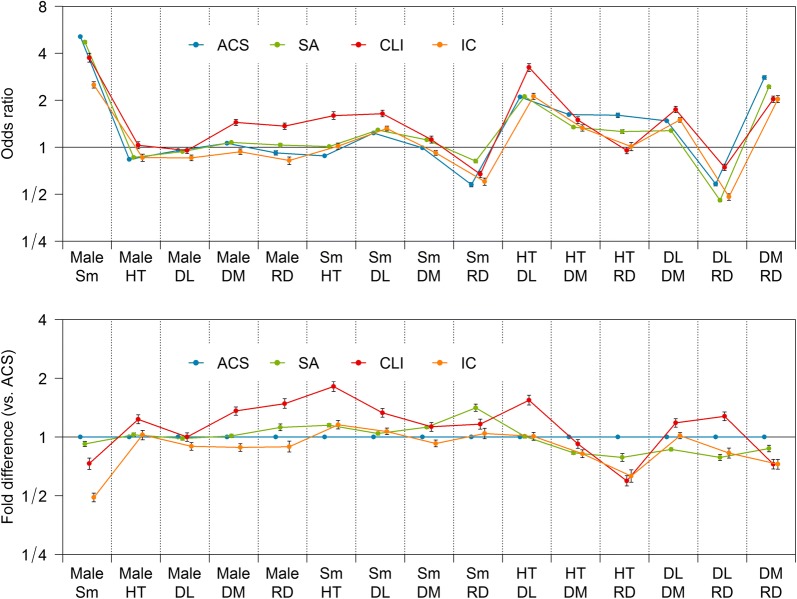
Likelihood of cardiovascular risk clustering in age-adjusted population. The upper panel shows the odds ratios of two arbitrary cardiovascular risk factors, quantifying the likelihood of the factors’ clustering, whereas the lower panel shows their fold difference relative to ACS. Error bars represent 95% CIs. *ACS* acute coronary syndrome, *SA* stable angina, *CLI* critical limb ischemia, *IC* intermittent claudication, *DL* dyslipidemia, *DM* diabetes mellitus, *HT* hypertension, *Male* male sex, *RD* end-stage renal disease on dialysis, *Sm* smoking

The likelihood of cardiovascular risk clustering also varied among CVDs (Fig. 2). For example, males were more frequently complicated with diabetes mellitus and end-stage renal disease on dialysis than females in a CLI population; however, such trends were not observed in an ACS population. Furthermore, smoking, hypertension, and dyslipidemia were more likely clustered with one another in a CLI population than in an ACS population. On the other hand, cases on dialysis were more frequently complicated with hypertension and diabetes mellitus than dialysis-free cases in an ACS population, whereas the trend was not obvious and less marked, respectively, in a CLI population.

**Fig. 3:**
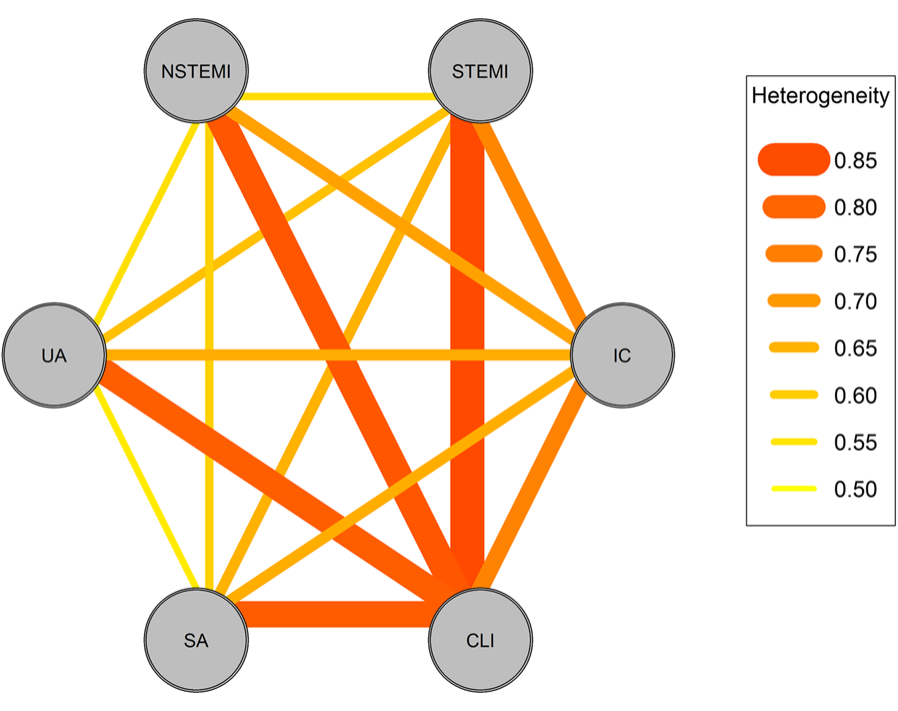
Heterogeneity in patient clinical profiles among CVDs. Data are the *C* statistics for heterogeneity in patient clinical profiles between CVDs. Their 95% CIs are presented in Additional file 1: Table S4. *CVD* cardiovascular diseases, *CLI* critical limb ischemia, *IC* intermittent claudication, *STEMI* ST-elevation myocardial infarction, *NSTEMI* non-STEMI, *UA* unstable angina pectoris, *SA* stable angina

Figure 3 and Additional file 1: Table S4 summarize the *C* statistics for heterogeneity in patient clinical profile between CVDs. LE-PAD and CAD demonstrated considerable heterogeneity; the heterogeneity was evident especially between CLI and ACS, with the *C* statistic equal to 0.833 (95% CI: 0.831–0.836), and further evident when compared STEMI and CLI with below-the-knee lesions (*C* statistic: 0.886 [0.884–0.888]).

Supplementary analyses with adjustment for the inter-institution variability demonstrated similar findings, except that the prevalence of end-stage renal disease on dialysis was relatively low compared to the primary analyses (Additional file 1: Tables S5–S7, Figures S1, S2).

## Discussion

The current study, based on analysis of data from nationwide procedural databases, describes comparative features of symptomatic LE-PAD and CAD warranting revascularization. As summarized by the *C* statistics for heterogeneity, patient profiles were not identical, but rather quite different between LE-PAD and CAD, especially between CLI and ACS. Given that currently major and familiar cardiovascular diseases in clinical practice are CAD rather than LE-PAD [16], a clearer understanding of the distinctive features of LE-PAD would be promoted by the current comparative analysis of LE-PAD versus CAD.

Compared to CAD, diabetes mellitus was more prevalent in LE-PAD, especially in CLI. As reported previously, diabetes mellitus increases the risk of lower-limb amputation [17, 18]. The high prevalence of diabetes mellitus in LE-PAD, especially in CLI, would confirm its high population attributable fraction for the limb prognosis. Other remarkable differences between CVDs included advanced age and end-stage renal disease on dialysis, both of which were again more frequently seen in LE-PAD. Diabetes mellitus, advanced age, and end-stage renal disease on dialysis are often recognized as major risk factors of perioperative morbidity in clinical practice [19–23]. Compared to PCI, lower-extremity EVT was likely targeted at a generally higher-risk population. Note that the prevalence of cardiovascular risk factors was different among CVDs even after stratification by age, indicating that their inter-CVD difference was not solely attributable to the difference in age distribution. The apparently lowered prevalence of end-stage renal disease on dialysis after adjustment for the inter-institution variability would simply come from the fact that patients on dialysis likely undergo interventions at a facility with a dialysis center. The comorbidity is clinically so closely linked with the institutional feature that the adjustment for the institution variable would statistically attenuate its prevalence.

In addition to the overall prevalence of cardiovascular risk factors, inter-CVD differences were observed in their clustering (Fig. 2). For example, the clustering of diabetes mellitus and end-stage renal disease on dialysis was more marked in an ACS population than in a CLI population, whereas the overall prevalence of these two cardiovascular risk factors per se was much higher in a CLI population. These findings suggest that differences in overall prevalence of cardiovascular risk factors between CVDs would not have any relationships with those in their clustering. Clinical guidelines have recommended cardiovascular risk management in LE-PAD patients, sometimes based on evidence shown in CAD patients, to cover scanty evidence in the field of LE-PAD [1, 2, 6, 7]. However, beneficial effects of an intervention to a cardiovascular risk factor would potentially vary according to the coexistence of another cardiovascular risk factor [8–12]. Furthermore, the efficacy and safety of some medications for a cardiovascular risk factor would be influenced by the coexistence of another cardiovascular risk factor, especially end-stage renal disease [24, 25]. The different cardiovascular risk clustering suggests that beneficial effects of the cardiovascular risk management including administration of certain medications in LE-PAD patients might be different from those expected from the studies in CAD patients.

The current analysis on patient profiles clarified another two features. First, patient profiles further varied with the vascular territory of LE-PAD (i.e., aortoiliac, femoropopliteal, and below-the-knee), even in the same clinical phenotypes (i.e., intermittent claudication or CLI). The association between treated vascular territories and patient profile in LE-PAD undergoing EVT was already reported [26, 27]. Those reports, however, did not divide LE-PAD into intermittent claudication and CLI. Given that below-the-knee revascularization is usually performed for CLI [28], treated vascular territories are expected to be strongly confounded by clinical phenotypes of LE-PAD. The current study, in which intermittent claudication and CLI were analyzed separately, successfully validated the previous findings that diabetes mellitus and end-stage renal disease on dialysis were associated with more distal arterial lesions, whereas smoking and dyslipidemia were associated with more proximal lesions, and that male versus female sex was related to aortoiliac and below-the-knee versus femoropopliteal lesions. On the other hand, some findings were not consistent with the previous reports [26, 27]: CLI with below-the-knee lesions had younger age and lower prevalence of hypertension than that with femoropopliteal lesions. Lower prevalence of hypertension was also seen in intermittent claudication with aortoiliac versus femoropopliteal lesions. The inconsistency might have come from our stratification by the clinical phenotypes, and our much larger sample size.

Second, younger patients were in general more likely exposed to cardiovascular risk factors than older patients in the LE-PAD population. When discussing the clinical impact of LE-PAD in the ageing society, some are apt to assume that cardiovascular risk factors are highly prevalent among seniors with LE-PAD [29]. The assumption would presumably lie on a general recognition that the risk of developing cardiovascular risk factors is commonly increased by age in a general population. The current findings might be paradoxical in this sense, but would be simply explained by the fact that, whereas age is a major promoter of atherosclerosis, the development of atherosclerosis (or vascular aging) is accelerated by cardiovascular risk factors. Patients with accumulated cardiovascular risk factors will develop LE-PAD earlier (i.e., at younger age), whereas those with fewer will develop the disease later (i.e., at older age). This would explain why age had an inverse correlation with the exposure to cardiovascular risk factors in the LE-PAD population.

One should note that the aim of this cross-sectional study was to compare clinical profile between an LE-PAD population and a CAD population, and not to assess risk factors of developing the CVDs and their prediction. The current findings of different clinical profiles might be suggestive of different developmental mechanisms between LE-PAD and CAD; however, the current study was only a cross-sectional and observational one, and underlying pathogeneses remained unrevealed. For example, we found the inter-CVD differences in prevalence and clustering of cardiovascular risk factors with adjustment for age. However, these findings only demonstrated the cross-sectional associations, and did not mean that the time course of development was adjusted. There still remained a possibility that ageing would be intermediating these apparent disparate findings, when one focuses on developmental mechanisms of the diseases. Moreover, the observed difference in clinical profile between CVDs might be biased and explained by other unmeasured confounders. Future studies are needed to uncover the underlying mechanisms of the differences between the CVDs.

The study had some other limitations. First, the J-PCI and J-EVT collect limited information regarding treated lesions and clinical profile. The databases included the data on whether individual cardiovascular risk factors were present or not, but not any biological data indicating their severity, such as blood pressure, serum lipids levels, plasma glucose levels, and serum creatinine levels to estimate glomerular filtration rate, all of which were previously reported to be associated with the risk of CVDs [30–33]. Neither were data on treatments or medications included. Furthermore, data on complications related to cardiovascular risk factors [34, 35], detailed severity of CVDs (e.g., foot infection in CLI [18]), and pathological lesion characteristics [36, 37] were also unavailable, and therefore their potential impact on inter-CVD differences remained to be explored. Second, smoking was only assessed by the definition as any history of smoking within the past 1 year, and the data were not available on smoking status otherwise defined (e.g., current smoking defined by present or prior smoking during the last 3 years) or quantitative measurements of smoking exposure. Third, the registries did not collect data on which diagnostic criteria of respective cardiovascular risk factors patients met. For example, although plasma glucose levels 2 h after a 75-g oral glucose tolerance test were included in the definition of diabetes mellitus, how many patients were really defined as diabetes mellitus by this criterion was unknown. Similarly, hypercholesterolemia and hypertriglyceridemia were not distinguished in dyslipidemia, although these two entities would not have the same effect in LE-PAD and CAD. Fourth, data were not available on arterial lesions coexisting but not indicated for revascularization. Fifth, the current study analyzed PCI and EVT cases in Japan. It remained unknown to what extent regional or racial differences would influence the findings [38]. Sixth, the current study analyzed cases undergoing percutaneous intervention, and did not target those undergoing open bypass surgery or those undergoing primary amputation. Clinical profiles might be different between the two revascularization procedures, although recent clinical studies have shown that major patient backgrounds are not so largely different between them in clinical practice [39, 40]. On the other hand, previous studies suggested that patient attributes would be different between LE-PAD patients undergoing revascularization and those undergoing primary amputation [41]. Future studies in other countries will be needed to validate the current findings.

## Conclusions

LE-PAD undergoing EVT, and CAD undergoing PCI had different patterns of age distribution and exposure to cardiovascular risk factors. The current study, which outlined comparative data of LE-PAD versus CAD, clarified distinguishing features of LE-PAD undergoing EVT versus CAD undergoing PCI at cardiovascular centers.

## Supplementary information

**Additional file 1.** Additional tables and figures.

## Data Availability

The data that support the findings of this study are available from the National Clinical Database, and the University hospital Medical Information Network, but restrictions apply to the availability of these data, which were used under license for the current study, and so are not publicly available. Data are however available from the authors upon reasonable request and with permission of the Japanese Association of Cardiovascular Intervention and Therapeutics, the National Clinical Database, and the University hospital Medical Information Network, in addition to the approval by the ethics committee of the Clinical Research Promotion Network Japan.

## Abbreviations

ACS: acute coronary syndrome
AMI: acute myocardial infarction
CAD: coronary artery disease
CI: confidence interval
CLI: critical limb ischemia
CVD: cardiovascular disease
EVT: endovascular therapy
LE-PAD: lower-extremity peripheral artery disease
PCI: percutaneous coronary intervention
SD: standard deviation
STEMI: ST- elevation myocardial infarction
