# Supplementary material for: Diabetes mellitus and other cardiovascular risk factors in lower-extremity peripheral artery disease versus coronary artery disease: an analysis of 1,121,359 cases from the nationwide databases
Source: Cardiovasc Diabetol. 2019 Nov 15;18:155. doi: 10.1186/s12933-019-0955-5 (PMC6857236; doi:10.1186/s12933-019-0955-5)
Supplement: Supplementary file 1 — Additional file 1. Additional tables and figures. [file 12933_2019_955_MOESM1_ESM.pdf]

Additional file 1: Table S1. Clinical profile compared to STEMI

	Age (years)	Age ≥75 years	Male sex	Smoking
STEMI	68.3±12.8 (Ref)	34.3% (Ref)	76.2% (Ref)	39.3% (Ref)
NSTEMI	69.8±12.4 (+1.5 [1.4 to 1.6])	39.2% (1.23 [1.21 to 1.26])	75.6% (0.96 [0.94 to 0.98])	35.8% (0.86 [0.85 to 0.88])
UA	70.6±11.2 (+2.3 [2.2 to 2.3])	40.0% (1.28 [1.26 to 1.29])	73.7% (0.87 [0.86 to 0.88])	29.2% (0.64 [0.63 to 0.65])
SA	70.7±10.1 (+2.4 [2.3 to 2.4])	38.7% (1.21 [1.19 to 1.22])	75.1% (0.94 [0.93 to 0.95])	26.6% (0.56 [0.55 to 0.56])
CLI	74.4±10.1 (+6.1 [6.0 to 6.2])	52.3% (2.10 [2.06 to 2.15])	65.6% (0.59 [0.58 to 0.61])	26.5% (0.56 [0.54 to 0.57])
IC	73.0±8.7 (+4.6 [4.5 to 4.7])	45.2% (1.58 [1.55 to 1.60])	75.6% (0.96 [0.94 to 0.98])	37.9% (0.94 [0.93 to 0.96])

Data are mean ± SD (difference versus STEMI [95% confidence interval]) for continuous variables and percentage (odds ratio versus STEMI [95% confidence interval]) for dichotomous variables. STEMI, ST-elevation myocardial infarction; NSTEMI, non-STEMI; UA, unstable angina pectoris; SA, stable angina; CLI, critical limb ischemia; IC, intermittent claudication.

Additional file 1: Table S1. Clinical profile compared to STEMI (continued)

	Hypertension	Dyslipidemia	Diabetes mellitus	End-stage renal disease on dialysis
STEMI	66.0% (Ref)	55.2% (Ref)	34.6% (ref)	2.0% (ref)
NSTEMI	71.5% (1.29 [1.26 to 1.32])	59.3% (1.18 [1.16 to 1.20])	39.1% (1.22 [1.19 to 1.24])	4.3% (2.16 [2.05 to 2.27])
UA	74.2% (1.48 [1.46 to 1.50])	62.1% (1.33 [1.31 to 1.34])	41.3% (1.33 [1.32 to 1.35])	6.9% (3.58 [3.46 to 3.70])
SA	76.5% (1.67 [1.65 to 1.69])	64.1% (1.45 [1.43 to 1.46])	44.6% (1.52 [1.51 to 1.54])	6.8% (3.52 [3.41 to 3.63])
CLI	73.0% (1.39 [1.36 to 1.43])	38.4% (0.51 [0.49 to 0.52])	65.5% (3.59 [3.52 to 3.67])	45.5% (40.1 [38.8 to 41.5])
IC	80.7% (2.16 [2.11 to 2.21])	55.5% (1.01 [0.99 to 1.03])	52.7% (2.11 [2.07 to 2.15])	14.9% (8.41 [8.11 to 8.72])

Data are mean ± SD (difference versus STEMI [95% confidence interval]) for continuous variables and percentage (odds ratio versus STEMI [95% confidence interval]) for dichotomous variables. STEMI, ST-elevation myocardial infarction; NSTEMI, non-STEMI; UA, unstable angina pectoris; SA, stable angina; CLI, critical limb ischemia; IC, intermittent claudication.

Additional file 1: Table S2. Clinical profile in LE-PAD by vessel territories

		Age (years)	Age ≥75 years	Male sex	Smoking
CLI	AI	74.9±9.9 (-0.2 [-0.5 to 0.1])	53.6% (0.95 [0.90 to 1.01])	70.9% (1.51 [1.42 to 1.60])	35.2% (1.48 [1.39 to 1.57])
	FP	75.1±10.0 (Ref)	54.9% (Ref)	61.8% (Ref)	26.8% (Ref)
	BK	74.1±10.2 (-1.0 [-1.2 to -0.8])	51.2% (0.86 [0.83 to 0.90])	66.0% (1.20 [1.15 to 1.24])	23.7% (0.85 [0.82 to 0.89])
IC	AI	72.6±8.6 (-0.8 [-0.9 to -0.6])	42.9% (0.84 [0.82 to 0.87])	82.3% (1.85 [1.78 to 1.92])	41.7% (1.29 [1.25 to 1.33])
	FP	73.3±8.7 (Ref)	47.1% (Ref)	71.6% (Ref)	35.6% (Ref)

Data are mean ± SD (difference versus FP [95% confidence interval]) for continuous variables and percentage (odds ratio versus FP [95% confidence interval]) for dichotomous variables. LE-PAD, lower-extremity peripheral artery disease; CLI, critical limb ischemia; IC, intermittent claudication; AI, aortoiliac artery; FP, femoropopliteal artery; BK, below-the-knee artery.

Additional file 1: Table S2. Clinical profile in LE-PAD by vessel territories (continued)

		Hypertension	Dyslipidemia	Diabetes mellitus	End-stage renal disease on dialysis
CLI	AI	75.7% (1.04 [0.97 to 1.11])	41.6% (1.08 [1.02 to 1.14])	55.6% (0.65 [0.61 to 0.69])	27.4% (0.53 [0.50 to 0.56])
	FP	74.9% (Ref)	39.8% (Ref)	65.9% (Ref)	41.7% (Ref)
	BK	71.0% (0.82 [0.79 to 0.85])	36.3% (0.86 [0.83 to 0.89])	68.4% (1.12 [1.08 to 1.16])	54.6% (1.68 [1.62 to 1.74])
IC	AI	79.9% (0.90 [0.86 to 0.93])	55.9% (1.03 [1.00 to 1.06])	46.4% (0.65 [0.63 to 0.67])	11.5% (0.61 [0.59 to 0.64])
	FP	81.6% (Ref)	55.1% (Ref)	57.0% (ref)	17.4% (ref)

Data are mean ± SD (difference versus FP [95% confidence interval]) for continuous variables and percentage (odds ratio versus FP [95% confidence interval]) for dichotomous variables. LE-PAD, lower-extremity peripheral artery disease; CLI, critical limb ischemia; IC, intermittent claudication; AI, aortoiliac artery; FP, femoropopliteal artery; BK, below-the-knee artery.

Additional file 1: Table S3. Association of cardiovascular risk factors with age

	ACS	SA	CLI	IC
(Intercept)	75.4 [75.4 to 75.5]	74.9 [74.8 to 74.9]	80.1 [79.9 to 80.4]	75.4 [75.2 to 75.6]
Male sex	-5.9 [-5.9 to -5.8]	-3.9 [-4.0 to -3.9]	-2.7 [-2.9 to -2.5]	-0.5 [-0.6 to -0.3]
Smoking	-5.9 [-5.9 to -5.8]	-3.3 [-3.4 to -3.2]	-3.5 [-3.7 to -3.3]	-3.2 [-3.3 to -3.1]
Hypertension	+3.3 [3.2 to 3.4]	+2.2 [2.1 to 2.2]	+1.8 [1.6 to 2.0]	+1.8 [1.6 to 1.9]
Dyslipidemia	-3.1 [-3.2 to -3.0]	-2.3 [-2.4 to -2.3]	-1.2 [-1.4 to -1.0]	-1.2 [-1.3 to -1.1]
Diabetes mellitus	-0.1 [-0.2 to -0.0]	-0.7 [-0.7 to -0.6]	-2.9 [-3.1 to -2.7]	-1.9 [-2.0 to -1.7]
End-stage renal disease on dialysis	-1.4 [-1.6 to -1.3]	-2.8 [-2.9 to -2.7]	-4.2 [-4.4 to -4.0]	-4.2 [-4.4 to -4.1]

Data are regression coefficients and their 95% confidence intervals, derived from multiple linear regression model in which age was entered as the dependent variable and cardiovascular risk factors (male sex, smoking, hypertension, dyslipidemia, diabetes mellitus, and end-stage renal disease on dialysis) as the dependent variables. ACS, acute coronary syndrome; SA, stable angina; CLI, critical limb ischemia; IC, intermittent claudication.

Additional file 1: Table S4. **Heterogeneity in clinical profile between CVDs.**

Cardiovascular diseases		*C* statistic
CAD	PAD	0.725 [0.723 to 0.726]
CAD	CLI	0.825 [0.823 to 0.827]
CAD	IC	0.673 [0.671 to 0.675]
CAD	CLI-AI	0.755 [0.749 to 0.761]
CAD	CLI-FP	0.821 [0.818 to 0.824]
CAD	CLI-BK	0.860 [0.857 to 0.862]
CAD	IC-AI	0.665 [0.662 to 0.668]
CAD	IC-FP	0.695 [0.692 to 0.697]
PAD	ACS	0.740 [0.738 to 0.742]
PAD	STEMI	0.773 [0.771 to 0.775]
PAD	NSTEMI	0.729 [0.726 to 0.731]
PAD	UA	0.713 [0.711 to 0.715]
PAD	SA	0.716 [0.714 to 0.717]
ACS	SA	0.595 [0.594 to 0.596]
ACS	CLI	0.833 [0.831 to 0.836]
ACS	IC	0.695 [0.693 to 0.697]
ACS	CLI-AI	0.762 [0.756 to 0.768]
ACS	CLI-FP	0.829 [0.827 to 0.832]
ACS	CLI-BK	0.867 [0.865 to 0.870]
ACS	IC-AI	0.683 [0.680 to 0.686]
ACS	IC-FP	0.717 [0.714 to 0.719]
STEMI	NSTEMI	0.567 [0.564 to 0.569]
STEMI	UA	0.621 [0.619 to 0.622]
STEMI	SA	0.651 [0.650 to 0.653]
STEMI	CLI	0.855 [0.853 to 0.857]
STEMI	IC	0.735 [0.733 to 0.737]
STEMI	CLI-AI	0.787 [0.781 to 0.792]
STEMI	CLI-FP	0.852 [0.849 to 0.854]
STEMI	CLI-BK	0.886 [0.884 to 0.888]
STEMI	IC-AI	0.719 [0.716 to 0.722]
STEMI	IC-FP	0.756 [0.754 to 0.759]
NSTEMI	UA	0.560 [0.558 to 0.563]
NSTEMI	SA	0.591 [0.589 to 0.594]
NSTEMI	CLI	0.829 [0.826 to 0.831]
NSTEMI	IC	0.682 [0.679 to 0.685]

(Continued)

NSTEMI	CLI-AI	0.752 [0.746 to 0.759]
NSTEMI	CLI-FP	0.824 [0.820 to 0.827]
NSTEMI	CLI-BK	0.864 [0.861 to 0.867]
NSTEMI	IC-AI	0.670 [0.667 to 0.674]
NSTEMI	IC-FP	0.704 [0.700 to 0.707]
UA	SA	0.541 [0.540 to 0.543]
UA	CLI	0.815 [0.813 to 0.817]
UA	IC	0.662 [0.660 to 0.664]
UA	CLI-AI	0.743 [0.737 to 0.749]
UA	CLI-FP	0.810 [0.807 to 0.813]
UA	CLI-BK	0.850 [0.847 to 0.853]
UA	IC-AI	0.658 [0.655 to 0.661]
UA	IC-FP	0.681 [0.679 to 0.684]
SA	CLI	0.820 [0.818 to 0.822]
SA	IC	0.659 [0.657 to 0.662]
SA	CLI-AI	0.753 [0.747 to 0.759]
SA	CLI-FP	0.816 [0.813 to 0.819]
SA	CLI-BK	0.854 [0.851 to 0.856]
SA	IC-AI	0.656 [0.653 to 0.659]
SA	IC-FP	0.679 [0.677 to 0.682]
CLI	IC	0.744 [0.741 to 0.747]
CLI	IC-AI	0.785 [0.782 to 0.789]
CLI	IC-FP	0.719 [0.716 to 0.723]
IC	CLI-AI	0.646 [0.639 to 0.654]
IC	CLI-FP	0.737 [0.733 to 0.741]
IC	CLI-BK	0.791 [0.788 to 0.794]
CLI-AI	CLI-FP	0.623 [0.615 to 0.631]
CLI-AI	CLI-BK	0.689 [0.682 to 0.697]
CLI-AI	IC-AI	0.685 [0.677 to 0.692]
CLI-AI	IC-FP	0.629 [0.622 to 0.637]
CLI-FP	IC-AI	0.782 [0.778 to 0.786]
CLI-FP	IC-FP	0.709 [0.705 to 0.714]
CLI-BK	IC-AI	0.828 [0.824 to 0.831]
CLI-BK	IC-FP	0.769 [0.765 to 0.772]
IC-AI	IC-FP	0.612 [0.608 to 0.617]

Data are *C* statistics [95% confidence intervals]. Some of the point estimates (but not 95% confidence intervals) are also illustrated in **Figure 3**. IC-AI and IC-FP indicate IC with AI and FP lesions undergoing EVT, whereas CLI-AI, CLI-FP, CLI-BK indicate CLI with AI, FP, and BK lesions undergoing EVT, respectively. CAD, coronary artery disease; PAD, peripheral artery disease; ACS, acute coronary syndrome; STEMI, ST-elevation myocardial infarction; NSTEMI, non-STEMI; UA, unstable angina; SA, stable angina; CLI, critical limb ischemia; IC, intermittent claudication, AI, aortoiliac artery; FP, femoropopliteal artery; BK, below-the-knee artery; EVT, endovascular therapy.

Additional file 1: Table S5. Institution-adjusted clinical profile

			Age (years)	Age ≥75 years	Male sex	Smoking
Model 1	CAD		70.3 [70.2 to 70.4] (Ref)	38.8% [38.4% to 39.1%] (Ref)	74.8% [74.5% to 75.0%] (Ref)	28.8% [28.0% to 29.7%] (Ref)
	LE-PAD		73.6 [73.5 to 73.8] (+3.4 [3.3 to 3.4])	48.5% [48.0% to 49.0%] (1.49 [1.47 to 1.51])	71.7% [71.3% to 72.1%] (0.85 [0.84 to 0.87])	32.0% [31.1% to 33.0%] (1.16 [1.15 to 1.18])
Model 2	ACS		69.7 [69.6 to 69.8] (Ref)	38.0% [37.6% to 38.4%] (Ref)	74.8% [74.6% to 75.1%] (Ref)	32.3% [31.4% to 33.3%] (Ref)
	SA		71.0 [70.9 to 71.1] (+1.3 [1.3 to 1.4])	39.6% [39.3% to 40.0%] (1.07 [1.06 to 1.08])	74.7% [74.4% to 75.0%] (0.99 [0.98 to 1.00])	24.8% [24.0% to 25.6%] (0.69 [0.68 to 0.69])
	CLI		74.7 [74.5 to 74.8] (+5.0 [4.9 to 5.1])	53.3% [52.7% to 54.0%] (1.86 [1.82 to 1.90])	65.6% [65.0% to 66.1%] (0.64 [0.63 to 0.66])	23.7% [22.9% to 24.6%] (0.65 [0.64 to 0.67])
	IC		73.1 [73.0 to 73.2] (+3.5 [3.4 to 3.5])	45.8% [45.2% to 46.3%] (1.38 [1.35 to 1.40])	75.4% [75.0% to 75.8%] (1.03 [1.01 to 1.05])	36.7% [35.6% to 37.7%] (1.21 [1.19 to 1.23])
Model 3	STEMI		68.7 [68.6 to 68.8] (Ref)	35.4% [35.0% to 35.8%] (Ref)	75.6% [75.3% to 75.9%] (Ref)	36.0% [35.0% to 37.0%] (Ref)
	NSTEMI		70.2 [70.0 to 70.3] (+1.5 [1.4 to 1.6])	40.5% [40.0% to 41.1%] (1.24 [1.22 to 1.27])	74.8% [74.4% to 75.3%] (0.96 [0.94 to 0.98])	32.6% [31.6% to 33.6%] (0.86 [0.84 to 0.88])
	UA		70.7 [70.6 to 70.8] (+2.0 [1.9 to 2.1])	40.3% [39.9% to 40.7%] (1.23 [1.22 to 1.25])	73.8% [73.5% to 74.1%] (0.91 [0.90 to 0.92])	28.2% [27.3% to 29.1%] (0.70 [0.69 to 0.71])
	SA		71.0 [70.9 to 71.1] (+2.3 [2.2 to 2.4])	39.6% [39.2% to 40.0%] (1.20 [1.18 to 1.21])	74.7% [74.4% to 75.0%] (0.95 [0.94 to 0.97])	24.8% [24.0% to 25.6%] (0.59 [0.58 to 0.59])
	CLI		74.7 [74.5 to 74.8] (+6.0 [5.9 to 6.1])	53.3% [52.7% to 54.0%] (2.08 [2.04 to 2.13])	65.6% [65.0% to 66.1%] (0.61 [0.60 to 0.63])	23.8% [22.9% to 24.6%] (0.55 [0.54 to 0.57])
	IC		73.1 [73.0 to 73.2] (+4.5 [4.4 to 4.6])	45.8% [45.2% to 46.3%] (1.54 [1.51 to 1.57])	75.4% [75.0% to 75.8%] (0.99 [0.97 to 1.01])	36.7% [35.6% to 37.8%] (1.03 [1.01 to 1.05])
Model 4	CLI	AI	75.0 [74.6 to 75.4] (-0.3 [-0.7 to 0.1])	53.8% [51.7% to 55.9%] (0.92 [0.85 to 1.00])	72.2% [70.5% to 73.9%] (1.62 [1.49 to 1.76])	32.8% [30.1% to 35.5%] (1.44 [1.32 to 1.57])
		FP	75.3 [75.0 to 75.6] (Ref)	55.8% [54.2% to 57.3%] (Ref)	61.6% [60.3% to 63.0%] (Ref)	25.3% [23.3% to 27.3%] (Ref)
		BK	73.9 [73.6 to 74.2] (-1.4 [-1.6 to -1.1])	50.6% [49.1% to 52.2%] (0.81 [0.77 to 0.86])	69.4% [68.2% to 70.6%] (1.41 [1.34 to 1.49])	21.1% [19.4% to 23.0%] (0.79 [0.74 to 0.84])
Model 5	IC	AI	72.3 [72.1 to 72.4] (-1.0 [-1.1 to -0.8])	41.3% [40.3% to 42.3%] (0.81 [0.78 to 0.84])	83.6% [83.0% to 84.3%] (2.05 [1.96 to 2.14])	41.5% [39.5% to 43.6%] (1.34 [1.29 to 1.40])
		FP	73.2 [73.0 to 73.4] (Ref)	46.5% [45.6% to 47.5%] (Ref)	71.4% [70.6% to 72.2%] (Ref)	34.6% [32.7% to 36.5%] (Ref)

Data are estimated means [95% confidence intervals] (difference [95% confidence intervals]) for continuous variables and estimated percentages (odds ratio [95% confidence interval]) for dichotomous variables.

Additional file 1: Table S5. Institution-adjusted clinical profile (continued)

			Hypertension	Dyslipidemia	Diabetes mellitus	End-stage renal disease on dialysis
Model 1	CAD		73.7% [73.1% to 74.2%] (Ref)	61.8% [61.0% to 62.7%] (Ref)	41.2% [40.8% to 41.6%] (Ref)	3.8% [3.5% to 4.0%] (Ref)
	LE-PAD		77.9% [77.3% to 78.5%] (1.26 [1.24 to 1.28])	49.3% [48.4% to 50.3%] (0.60 [0.59 to 0.61])	56.8% [56.3% to 57.3%] (1.88 [1.85 to 1.90])	16.0% [15.2% to 17.0%] (4.89 [4.79 to 4.98])
Model 2	ACS		70.3% [69.7% to 70.9%] (Ref)	58.1% [57.3% to 59.0%] (Ref)	37.8% [37.4% to 38.2%] (Ref)	3.0% [2.8% to 3.2%] (Ref)
	SA		77.5% [77.0% to 78.1%] (1.46 [1.44 to 1.47])	66.0% [65.2% to 66.8%] (1.40 [1.39 to 1.41])	45.2% [44.7% to 45.6%] (1.35 [1.34 to 1.37])	4.8% [4.5% to 5.1%] (1.65 [1.62 to 1.68])
	CLI		73.7% [73.0% to 74.4%] (1.18 [1.15 to 1.21])	40.0% [39.0% to 41.1%] (0.48 [0.47 to 0.49])	64.8% [64.2% to 65.4%] (3.03 [2.96 to 3.10])	29.3% [27.9% to 30.7%] (13.5 [13.2 to 13.9])
	IC		81.0% [80.4% to 81.5%] (1.80 [1.76 to 1.84])	55.5% [54.5% to 56.5%] (0.90 [0.88 to 0.92])	52.5% [51.9% to 53.0%] (1.81 [1.78 to 1.85])	9.4% [8.8% to 10.0%] (3.38 [3.29 to 3.48])
Model 3	STEMI		65.9% [65.2% to 66.6%] (Ref)	54.4% [53.5% to 55.3%] (Ref)	34.5% [34.0% to 34.9%] (Ref)	1.4% [1.3% to 1.5%] (Ref)
	NSTEMI		71.1% [70.3% to 71.8%] (1.27 [1.25 to 1.30])	58.1% [57.1% to 59.0%] (1.16 [1.14 to 1.18])	38.8% [38.3% to 39.4%] (1.21 [1.19 to 1.23])	2.9% [2.7% to 3.1%] (2.08 [1.98 to 2.18])
	UA		75.3% [74.7% to 75.8%] (1.57 [1.55 to 1.60])	62.8% [61.9% to 63.7%] (1.42 [1.40 to 1.43])	41.4% [41.0% to 41.9%] (1.35 [1.33 to 1.36])	4.9% [4.6% to 5.2%] (3.66 [3.54 to 3.79])
	SA		77.5% [77.0% to 78.0%] (1.78 [1.76 to 1.80])	66.0% [65.1% to 66.8%] (1.63 [1.61 to 1.64])	45.1% [44.7% to 45.6%] (1.56 [1.55 to 1.58])	4.8% [4.5% to 5.1%] (3.55 [3.44 to 3.66])
	CLI		73.7% [73.0% to 74.4%] (1.45 [1.41 to 1.49])	40.0% [39.0% to 41.1%] (0.56 [0.55 to 0.57])	64.8% [64.2% to 65.4%] (3.50 [3.42 to 3.59])	29.4% [28.0% to 30.8%] (29.4 [28.3 to 30.6])
	IC		81.0% [80.5% to 81.6%] (2.21 [2.16 to 2.26])	55.5% [54.5% to 56.5%] (1.05 [1.03 to 1.07])	52.5% [51.9% to 53.1%] (2.10 [2.06 to 2.14])	9.4% [8.9% to 10.0%] (7.35 [7.07 to 7.64])
Model 4	CLI	AI	75.6% [73.6% to 77.5%] (0.93 [0.85 to 1.02])	41.1% [38.6% to 43.7%] (0.99 [0.91 to 1.07])	53.7% [51.6% to 55.9%] (0.59 [0.55 to 0.64])	18.1% [16.2% to 20.1%] (0.56 [0.50 to 0.61])
		FP	76.9% [75.4% to 78.4%] (Ref)	41.5% [39.3% to 43.7%] (Ref)	66.2% [64.7% to 67.7%] (Ref)	28.4% [26.3% to 30.7%] (Ref)
		BK	72.1% [70.4% to 73.7%] (0.77 [0.73 to 0.82])	36.9% [34.8% to 39.0%] (0.83 [0.78 to 0.87])	68.9% [67.4% to 70.3%] (1.13 [1.07 to 1.19])	44.1% [41.5% to 46.8%] (1.99 [1.88 to 2.10])
Model 5	IC	AI	79.9% [78.8% to 80.9%] (0.84 [0.81 to 0.88])	56.0% [54.3% to 57.7%] (1.01 [0.97 to 1.04])	45.0% [43.9% to 46.1%] (0.58 [0.56 to 0.60])	7.5% [6.8% to 8.2%] (0.53 [0.50 to 0.56])
		FP	82.5% [81.6% to 83.4%] (Ref)	55.8% [54.2% to 57.4%] (Ref)	58.4% [57.5% to 59.4%] (Ref)	13.2% [12.1% to 14.3%] (Ref)

Data are estimated means [95% confidence intervals] (difference [95% confidence intervals]) for continuous variables and estimated percentages (odds ratio [95% confidence interval]) for dichotomous variables.

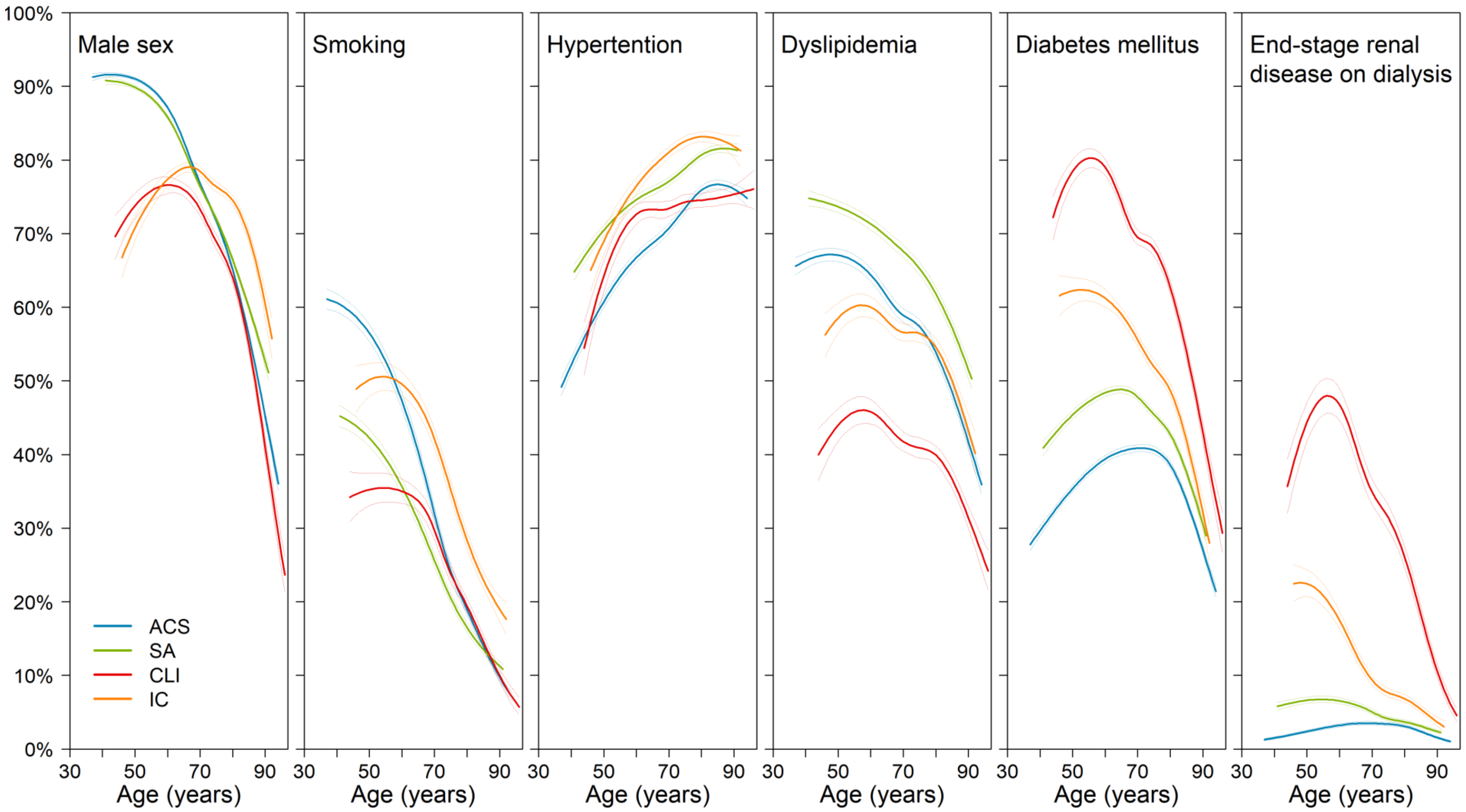

Additional file 1: Figure S1. Institution-adjusted prevalence of cardiovascular risk factors by age.

Solid lines and dotted lines represent estimates and their 95% CIs, corresponding to each age (mean–3SD to mean+3SD of age). ACS, acute coronary syndrome; SA, stable angina; CLI, critical limb ischemia; IC, intermittent claudication.

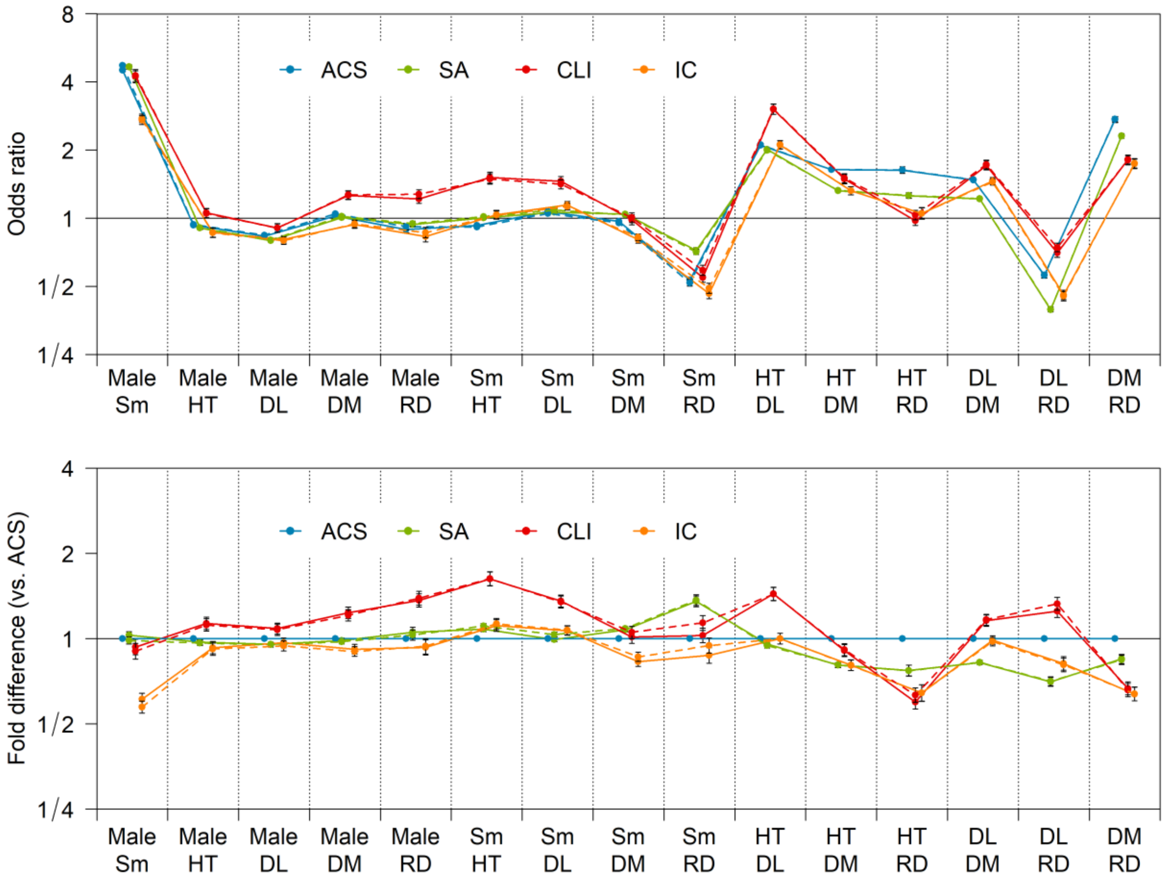

Additional file 1: Figure S2. Likelihood of cardiovascular risk clustering with adjustment for age and institution.

The upper panel shows the odds ratios of two arbitrary cardiovascular risk factors, quantifying the likelihood of the factors' clustering, whereas the lower panel shows their fold difference relative to ACS. Dots connected with solid lines indicates estimates calculated from the generalized linear mixed model with a logit-link function including a variable in the upper row as the dependent variable, another in the lower row and age as the fixed effects, and the inter-institution variability as the random effects. On the other hand, Dots connected with dotted lines indicates estimates calculated from the generalized linear mixed model with a logit-link function including a variable in the upper row as the dependent variable, another in the lower row and age as the fixed effects, and the inter-institution variability as the random effects. Note that both estimates were close to each other. Error bars represent 95% confidence intervals. ACS, acute coronary syndrome; SA, stable angina; CLI, critical limb ischemia; IC, intermittent claudication; DL, dyslipidemia; DM, diabetes mellitus; HT, hypertension; Male, male sex; RD, end-stage renal disease on dialysis; Sm, smoking.

Additional file 1: Table S6. Institution-adjusted association of cardiovascular risk factors with age

	ACS	SA	CLI	IC
(Intercept)	75.7 [75.6 to 75.8]	75.2 [75.1 to 75.3]	80.1 [79.9 to 80.4]	75.5 [75.2 to 75.8]
Male sex	-5.7 [-5.8 to -5.6]	-3.7 [-3.8 to -3.7]	-2.6 [-2.8 to -2.3]	-0.4 [-0.6 to -0.2]
Smoking	-6.0 [-6.1 to -6.0]	-3.6 [-3.7 to -3.5]	-3.6 [-3.8 to -3.4]	-3.4 [-3.6 to -3.2]
Hypertension	3.2 [3.2 to 3.3]	2.1 [2.0 to 2.1]	1.7 [1.5 to 1.9]	1.7 [1.5 to 1.9]
Dyslipidemia	-3.2 [-3.3 to -3.1]	-2.4 [-2.5 to -2.4]	-1.3 [-1.5 to -1.1]	-1.2 [-1.4 to -1.1]
Diabetes mellitus	-0.1 [-0.2 to -0.1]	-0.7 [-0.7 to -0.6]	-2.8 [-3.1 to -2.6]	-1.9 [-2.0 to -1.7]
End-stage renal disease on dialysis	-1.4 [-1.6 to -1.3]	-2.9 [-3.0 to -2.8]	-4.2 [-4.5 to -4.0]	-4.2 [-4.5 to -4.0]

Data are regression coefficients and their 95% confidence intervals, derived from the generalized linear mixed model including age as the dependent variable, cardiovascular risk factors (male sex, smoking, hypertension, dyslipidemia, diabetes mellitus, and end-stage renal disease on dialysis) as the fixed effects, and the inter-institution variability as the mixed effects. ACS, acute coronary syndrome; SA, stable angina; CLI, critical limb ischemia; IC, intermittent claudication.

Additional file 1: Table S7. **Institution-adjusted heterogeneity in clinical profile between CVDs.**

Cardiovascular diseases		*C* statistic
CAD	PAD	0.725 [0.723 to 0.726]
CAD	CLI	0.825 [0.823 to 0.827]
CAD	IC	0.673 [0.671 to 0.675]
CAD	CLI-AI	0.754 [0.748 to 0.760]
CAD	CLI-FP	0.821 [0.818 to 0.824]
CAD	CLI-BK	0.859 [0.857 to 0.862]
CAD	IC-AI	0.665 [0.661 to 0.668]
CAD	IC-FP	0.694 [0.692 to 0.697]
PAD	ACS	0.740 [0.739 to 0.742]
PAD	STEMI	0.773 [0.771 to 0.775]
PAD	NSTEMI	0.729 [0.726 to 0.731]
PAD	UA	0.712 [0.710 to 0.714]
PAD	SA	0.715 [0.714 to 0.717]
ACS	SA	0.595 [0.594 to 0.596]
ACS	CLI	0.833 [0.831 to 0.835]
ACS	IC	0.695 [0.693 to 0.697]
ACS	CLI-AI	0.762 [0.756 to 0.768]
ACS	CLI-FP	0.829 [0.826 to 0.832]
ACS	CLI-BK	0.867 [0.865 to 0.870]
ACS	IC-AI	0.683 [0.680 to 0.686]
ACS	IC-FP	0.717 [0.714 to 0.719]
STEMI	NSTEMI	0.567 [0.564 to 0.569]
STEMI	UA	0.619 [0.618 to 0.621]
STEMI	SA	0.650 [0.649 to 0.652]
STEMI	CLI	0.855 [0.853 to 0.857]
STEMI	IC	0.735 [0.733 to 0.737]
STEMI	CLI-AI	0.786 [0.781 to 0.792]
STEMI	CLI-FP	0.851 [0.849 to 0.854]
STEMI	CLI-BK	0.886 [0.883 to 0.888]
STEMI	IC-AI	0.720 [0.716 to 0.723]
STEMI	IC-FP	0.756 [0.754 to 0.759]
NSTEMI	UA	0.557 [0.555 to 0.560]
NSTEMI	SA	0.590 [0.588 to 0.593]
NSTEMI	CLI	0.828 [0.826 to 0.831]
NSTEMI	IC	0.682 [0.679 to 0.685]

(Continued)

NSTEMI	CLI-AI	0.752 [0.745 to 0.758]
NSTEMI	CLI-FP	0.823 [0.820 to 0.826]
NSTEMI	CLI-BK	0.864 [0.861 to 0.867]
NSTEMI	IC-AI	0.671 [0.667 to 0.675]
NSTEMI	IC-FP	0.704 [0.701 to 0.707]
UA	SA	0.540 [0.539 to 0.542]
UA	CLI	0.814 [0.812 to 0.817]
UA	IC	0.662 [0.659 to 0.664]
UA	CLI-AI	0.742 [0.736 to 0.749]
UA	CLI-FP	0.809 [0.806 to 0.812]
UA	CLI-BK	0.850 [0.847 to 0.852]
UA	IC-AI	0.658 [0.655 to 0.661]
UA	IC-FP	0.681 [0.678 to 0.683]
SA	CLI	0.820 [0.818 to 0.822]
SA	IC	0.658 [0.656 to 0.661]
SA	CLI-AI	0.752 [0.746 to 0.758]
SA	CLI-FP	0.816 [0.813 to 0.818]
SA	CLI-BK	0.854 [0.851 to 0.856]
SA	IC-AI	0.655 [0.652 to 0.658]
SA	IC-FP	0.678 [0.676 to 0.681]
CLI	IC	0.743 [0.740 to 0.746]
CLI	IC-AI	0.785 [0.781 to 0.788]
CLI	IC-FP	0.718 [0.715 to 0.721]
IC	CLI-AI	0.644 [0.637 to 0.652]
IC	CLI-FP	0.736 [0.732 to 0.740]
IC	CLI-BK	0.790 [0.787 to 0.793]
CLI-AI	CLI-FP	0.623 [0.615 to 0.631]
CLI-AI	CLI-BK	0.689 [0.682 to 0.696]
CLI-AI	IC-AI	0.684 [0.676 to 0.691]
CLI-AI	IC-FP	0.626 [0.619 to 0.634]
CLI-FP	IC-AI	0.781 [0.777 to 0.785]
CLI-FP	IC-FP	0.708 [0.704 to 0.713]
CLI-BK	IC-AI	0.827 [0.823 to 0.830]
CLI-BK	IC-FP	0.767 [0.764 to 0.771]
IC-AI	IC-FP	0.612 [0.608 to 0.617]

Data are *C* statistics [95% confidence intervals]. IC-AI and IC-FP indicate IC with AI and FP lesions undergoing endovascular therapy, whereas CLI-AI, CLI-FP, CLI-BK indicate CLI with AI, FP, and BK lesions undergoing endovascular therapy, respectively. CAD, coronary artery disease; PAD, peripheral artery disease; ACS, acute coronary syndrome; STEMI, ST-elevation myocardial infarction; NSTEMI, non-STEMI; UA, unstable angina; SA, stable angina; CLI, critical limb ischemia; IC, intermittent claudication, AI, aortoiliac artery; FP, femoropopliteal artery; BK, below-the-knee artery.
